# Acceptance of Virtual Reality Among Adults with Rheumatologic Conditions

**DOI:** 10.1089/jmxr.2024.0024

**Published:** 2024-12-18

**Authors:** Kimberly B. Garza, Heqin Yang, Alexicia Richardson, Cheryl Seals, Gary Hawkins, Chad G. Rose, Willliam B. Nowell, Kelly Gavigan, Patrick Stewart, Liana Fraenkel, Jeffrey R. Curtis

**Affiliations:** 1 Auburn University, Auburn, Alabama, USA.; 2 Global Healthy Living Foundation, Upper Nyack, New York, USA.; 3 ENRGY, Hoover, Alabama, USA.; 4 Berkshire Medical Center, Pittsfield, Massachusetts, USA.; 5 Yale University School of Medicine, New Haven, Connecticut, USA.; 6 University of Alabama at Birmingham, Birmingham, Alabama, USA.

**Keywords:** rheumatologic conditions, virtual reality, anticipated enjoyment, willingness to use, perceived usefulness

## Abstract

Risk communication to patients with rheumatologic conditions is difficult. Virtual reality (VR) has demonstrated utility as a delivery mechanism for patient education, but user acceptance of this technology for applications such as communicating risks and benefits of health care interventions and medical treatments has been poorly studied. We aimed to assess the acceptability of VR as an educational tool among people with rheumatologic conditions and to compare acceptability among those who had used VR in the past to those who are VR naïve. A cross-sectional, web-based survey was conducted, sampling two populations: members of an online patient-powered research network (registry) that included adults with rheumatologic conditions, and patients within a large U.S. community rheumatology practice-based research network. Survey items included patient demographic and clinical characteristics, prior VR experience, perceived usefulness for learning about their condition, anticipated enjoyment, and willingness to use VR to manage disease. A total of 3927 individuals completed the survey. Overall, 14.9% reported having prior experience using VR, and of these, 54.0% had used it only one to two times. Twenty-nine percent of participants agreed that VR would be useful for learning about their condition (25.4% of VR naïve, 49.0% of VR experienced [*p* < 0.001]), while the majority of participants (62.6%) were unsure (66.1% of VR naïve, 42.7% of VR experienced [*p* < 0.001]). More than one-third (38.9%) of participants agreed that they would enjoy using VR for this purpose (35.3% of VR naïve, 59.7% of VR experienced [*p* < 0.001]), while 47.8% were unsure (51.1% of VR naïve, 28.2% of VR experienced [*p* < 0.001]). Most study participants (86.0%) reported willingness to use VR to manage their disease, regardless of prior experience. Even among those with no prior experience using VR, people with rheumatologic conditions report acceptance of VR to manage their condition. These findings support further exploration of the potential value of interventions that introduce VR to this patient population for use in learning about and managing their disease.

## Introduction

Patients’ acceptance of health care provider recommendations to initiate and escalate effective medications is critical to achieving the best outcomes in rheumatic diseases.^1^ However, acceptance of treat-to-target recommendations and adherence to treatment is often low in this population.^2,3^ Discordance between rheumatologists’ recommendations and patients’ willingness to escalate therapy was found to contribute to risk of developing difficult-to-treat rheumatoid arthritis.^4^ As postulated by the Health Belief Model,^5^ inaccurate perceptions of risk and severity of disease progression and/or the perception that treatment benefits do not outweigh the short-term potential harms and costs can negatively impact acceptance of treatment recommendations. The consequences of poorly controlled rheumatologic diseases are difficult to demonstrate using traditional risk communication approaches. Providing balanced information is essential to effectively frame appropriate messaging and present both the risks of undertreatment and the benefits of treatment in a way that is salient and cognitively impactful to patients. Interventions to support communication between patients and rheumatologists have the potential to increase treatment acceptance and improve patient outcomes. Owensby et al. examined perceived barriers to achieving treat-to-target goals among patients with rheumatoid arthritis and rheumatologists and found that suboptimal patient–rheumatologist communication and perceived patient medication risk aversion were among the most frequently endorsed barriers that could likely be addressed through a behavioral intervention.^6^

Patients with rheumatic disease face the prospect of negative future impacts on daily functioning and quality of life. Fraenkel et al. found that high disease activity was associated with future escalation of therapy in patients with rheumatoid arthritis, but only when the perceived severity of consequences of disease progression was high.^7^ Educational interventions that help patients better understand how their condition may progress over time if inadequately treated may support their decision-making related to engagement in, and maintenance of, the recommended treatment plan. Virtual reality (VR) offers a novel solution to improve perceptions of the risks of uncontrolled rheumatologic diseases. Because of the realistic qualities of an immersive VR experience, consequences of the natural progression of disease presented in VR can have greater salience compared with verbal or textual presentation. By creating a sense of “being there” (presence), a sense that events in VR are actually happening (plausibility), and a sense that the body of a virtual avatar is one’s own body (body ownership), immersive VR can simulate realistic scenarios allowing the user to experience “first-hand” the effects of disease progression on physical functioning and quality of life.

Past research has demonstrated positive perceptions among the general public on the use of VR in health care,^8^ and as an intervention, VR has been shown to decrease pain and anxiety in patients with rheumatologic conditions.^9–11^ Additionally, VR serves as a valuable tool for conveying information to patients about their disease^12^ and for exercise training and rehabilitation.^13^ However, user acceptance of VR as an educational tool among people with rheumatologic conditions is unknown.

The technology acceptance model (TAM) has been widely used as a guiding theoretical framework for user acceptance.^14^ The TAM proposes that attitudes, including perceived usefulness and ease of use of a technology, influence end users’ intention to use it. This model applies broadly, encompassing both naïve users and those familiar with similar technologies. Naïve users may rely more heavily on their initial perceptions about the technology due to their limited experience. On the contrary, prior experiences with similar technologies may influence how experienced users perceive the new technology’s potential benefits, influencing their intentions to adopt it. In a study of user acceptance of computer technology that utilized the TAM as a framework, perceived usefulness was shown to explain more than 50% of the variance in intention to use, while perceived ease of use explained a smaller portion of the variance, with the effect of the latter subsiding over time.^14^ Sagnier et al. likewise found that perceived ease of use did not have a significant impact on intention to use VR after performing an assembly task in a study of psychology and engineering students.^15^ Thus, perceived usefulness is a key metric in the expectation that patients will engage with VR technology for health-related reasons. In another study among college students, enjoyment was shown to be positively associated with acceptance of a new computing technology when controlling for perceived usefulness.^16^ In a study by Mascret et al., perceived enjoyment was found to be the most powerful predictor of intention to use a VR headset to prevent falls among older adults.^17^

The objective of this study was to assess interest in the use of VR as an educational tool among a large sample of people with rheumatologic diseases by exploring the perceived usefulness of VR to learn about their condition, as well as their anticipated enjoyment of and willingness to use VR for this purpose. A secondary aim was to compare these constructs among participants who reported prior experience with VR versus those who reported having no experience.

## Methods

A cross-sectional web-based survey was conducted from January to June 2022 in two rheumatic disease populations: (1) members of ArthritisPower (AP), an online patient-powered research network (registry) that includes ∼13,000 adults with rheumatologic conditions^18^; and (2) patients within the Excellence Network in Rheumatology (ENRGY), a large U.S. community rheumatology practice-based research network (PBRN) consisting of patient data from more than 700 rheumatology providers, with identifiable data and direct access to ∼173,000 patients in the subgroup of more than 300 rheumatology providers. Participants were invited to participate in this survey if they participated in a separate educational priorities survey sent by email, which required that the participant be 19 years of age or older and have a diagnosis of at least one of the following conditions: ankylosing spondylitis or axial spondyloarthritis, fibromyalgia, gout, juvenile idiopathic arthritis, lupus, myositis, osteoarthritis, osteoporosis, polymyalgia rheumatica, psoriatic arthritis, rheumatoid arthritis (RA), Sjogren’s syndrome, or vasculitis, as diagnosed by a rheumatologist according to self-report in the patient registry, and based on diagnoses from rheumatology providers documented in the electronic health record in the PBRN. A total of 4598 people completed the previous educational priorities survey. All of them answered the question about whether they thought VR would help them and other people with their condition: 2110 responded “yes” and 1875 responded “not sure,” which resulted in 3985 people (87.7%) being directed to participate in this VR survey. We did not include those who responded “no” because their response implied a lack of interest in answering the survey questions related to VR.

The preamble of the survey contained a photograph of a person using a VR headset and controllers along with a brief description of the technology. Survey items included experience using VR, perceived usefulness and anticipated enjoyment of VR to learn about their condition, and willingness to use VR to manage their condition. Perceived usefulness and anticipated enjoyment were measured by asking, “Do you think you would learn useful information about your condition using virtual reality?” and “Do you think that you would enjoy using virtual reality to learn more about your condition?” with response options including “yes,” “no,” and “not sure.” Willingness to use VR was measured by asking, “In which of the following ways might you be willing to use VR in the management of your condition?” Select all that apply: “to play games,” “for exercise,” “for education,” “to interact with others who may have my condition,” “to interact with my healthcare provider,” “for physical therapy,” “for emotional therapy,” and “none of the above,” and coded as willing to use if the participant did not choose “none of the above.” Demographic survey items included gender and race, each using a fixed set of categories, and age using the date of birth. Rheumatologic conditions and year of diagnosis were also included. For the AP survey, a check-all-that-apply question asked about conditions, while in the ENRGY group, the primary rheumatologic diagnosis was requested, therefore prevalence of individual conditions could not be calculated across the entire sample. Descriptive statistics, independent samples *t*-test, and Pearson’s chi-square were used to characterize the sample and compare perceived usefulness, anticipated enjoyment, and willingness to use VR in people with and without prior VR experience. Frequencies were calculated by source due to potential differences in the two populations, an online community versus patients in a PBRN. Data were analyzed using IBM SPSS Version 27. The study was approved by a Central Institutional Review Board (Advarra), consent was obtained from all participants, registry data were de-identified, and researchers were given permission to use the data.

## Results

Of those 3985 eligible to participate, 3927 (98.5%) completed the survey (708 individuals from AP and 3219 from ENRGY). Mean age was 63 years, 80.7% of participants were female and 81.1% were White, 6.0% were black or African American, and 6.5% were Hispanic (Table 1). Among the ENRGY group, the most prevalent primary rheumatologic conditions according to the electronic health record were rheumatoid arthritis (39.3%), osteoarthritis (16.2%), and psoriatic arthritis (14.3%). More than half (56.6%) of participants in the AP group reported having RA, 55.1% reported having osteoarthritis, and 17.4% reported having psoriatic arthritis. A small portion (14.9%) of participants reported having any prior experience using VR, and of these, the majority (54.0%) had only used it one to two times. VR-experienced participants were younger (*p* < 0.001), a significantly lower proportion were white (*p* < 0.001), and a significantly greater proportion had received their diagnosis in the past 10 years (*p* < 0.001) compared with VR-naïve participants. There was no significant difference in the proportion of females among VR-experienced and VR-naïve participants (*p* = 0.055). Comparison of study participants to the patient populations of each recruitment source is shown in Supplementary Table S1.

**Table 1.** Characteristics of Survey Participants

	Total (*n* = 3927)^b^	VR naïve (*n* = 3341)	VR experienced (*n* = 585)	*p* value^a^
Age (in years) mean ± SD	62.7 ± 11.9	63.7 ± 11.6	57.1 ± 12.2	**<0.001**
Female *n* (%)	3168 (80.7%)	2712 (81.2%)	455 (77.8%)	0.055
Race *n* (%)				**0.032**
White *n* (%)	3184 (81.1%)	2731 (81.7%)	452 (77.3%)	
Black *n* (%)	236 (6.0%)	184 (5.5%)	52 (8.9%)	
Other *n* (%)	507 (12.9%)	426 (12.8%)	81 (13.8%)	
Hispanic *n* (%)	255 (6.5%)	225 (6.7%)	30 (5.1%)	**0.173**
Diagnosed in past 10 years *n* (%)	1960 (54.8%)	1636 (53.7%)	323 (60.8%)	**0.002**

a Calculated using independent samples *t*-test for mean age and chi-square for all other data.

b One respondent did not answer the question about prior experience.

VR, virtual reality; SD, standard deviation.

Note: Bold *p* values represent statistically significant comparisons.

### Perceived usefulness

**FIG. 1.**
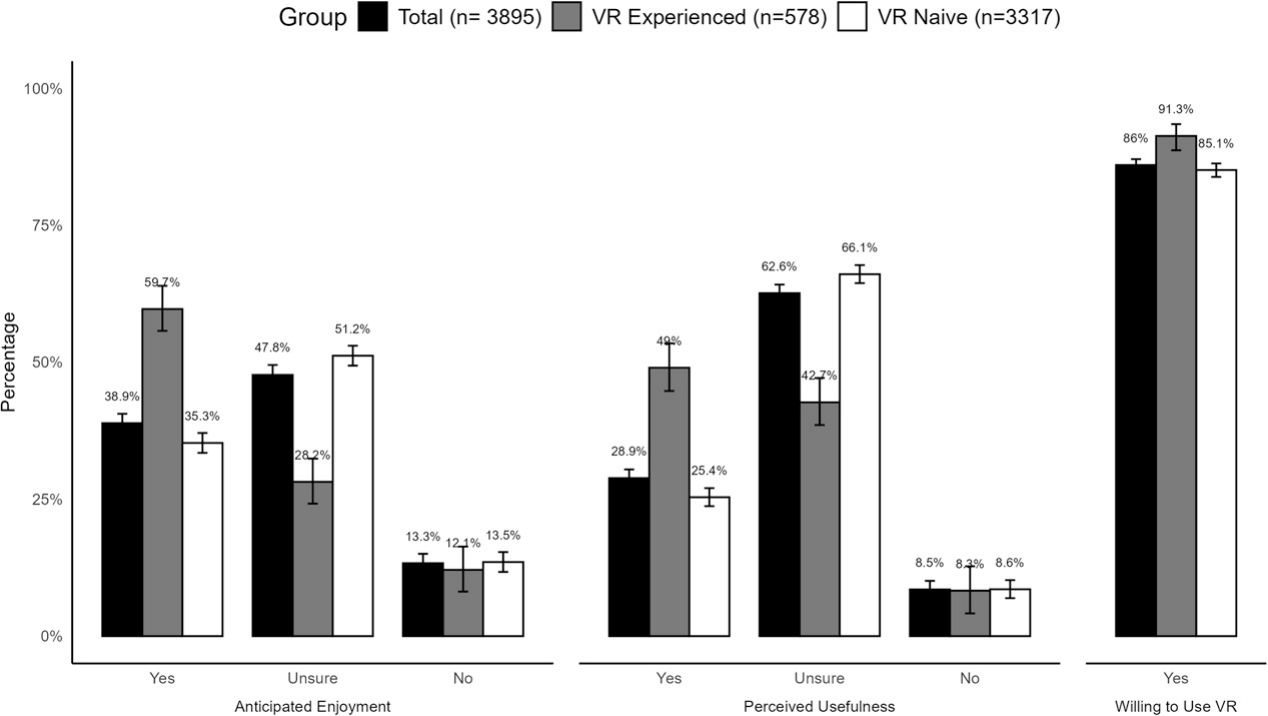
Perceived usefulness, anticipated enjoyment, and willingness to use VR to manage their health condition. ^a^*p* values were calculated using Pearson’s chi-square comparing “yes” responses to “unsure/no” responses for each of the three constructs. *p* < 0.001 for all comparisons. ^b^Willingness to use VR represents the number of participants who did not check “none of the above” for various VR use cases shown in Figure 2; there were fewer total participants (*n* = 3870) for this question due to missing data. VR, virtual reality.

Of those with no prior experience using VR, one-quarter (25.4%) thought VR would be useful to learn more about their condition, while the majority (66.1%) were unsure (Fig. 1). Of those with prior experience using VR, about half (49.0%) thought they could learn useful information about their condition using VR, with a greater proportion in the AP group (54.5%) compared with the ENRGY group (47.6%) (Supplementary Table S2). A significantly greater proportion of participants with VR experience thought that VR would be useful compared with those with no prior experience using VR (*p* < 0.001). Of the respondents to the prior educational priorities survey, 613 reported that they did not think VR would help them or other people with their condition, and these were excluded from the main analysis. However, if we instead assume that these respondents would also answer “no” to our question about perceived usefulness of VR to learn more about their condition, our new denominator for that survey item becomes 4508 and the adjusted frequencies would be: “yes” = 1125 (25.0%) (rather than 28.9%), “unsure” = 2438 (54.1%), and “no” = 945 (21.0%) for the full sample. This rate for perceived usefulness is only 4 percentage points lower than our original calculation.

**FIG. 2.**
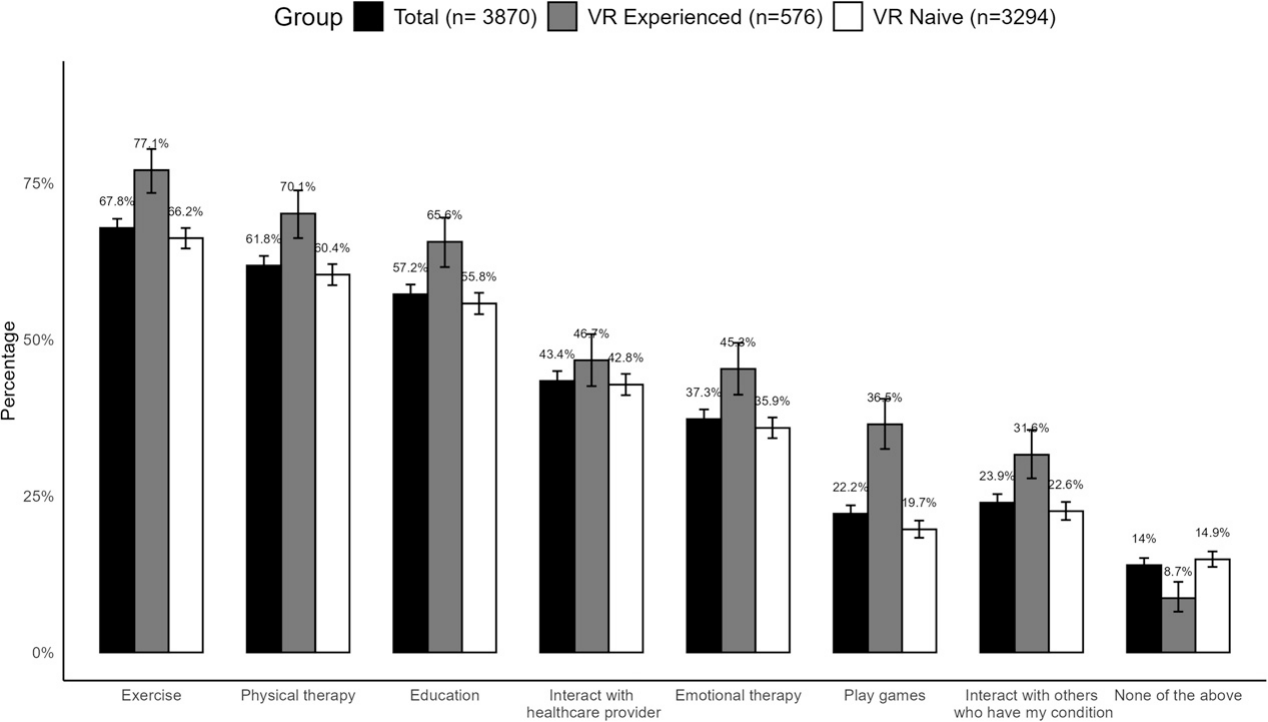
Ways people with rheumatologic conditions might be willing to use VR to manage their health conditions. ^a^Frequencies do not add up to 100% because multiple response options could be selected. ^b^*p* values for comparisons between VR naïve and VR experienced were calculated using Pearson’s chi-square. *p* < 0.001 for all categories except “to interact with my health care provider” which was *p* = 0.082.

### Anticipated enjoyment

Of those with no prior VR experience, about one-third (35.3%) thought they would enjoy using VR to learn more about their disease (Fig. 1). Comparing by recruitment source, 51.0% of AP participants and 31.9% of ENRGY participants reported anticipated enjoyment (Supplementary Table S2). Of those with prior VR experience, over half (59.7%) thought they would enjoy using VR for this purpose. This was higher in AP participants (73.2%) compared with ENRGY participants (56.4%). A significantly greater proportion of participants with VR experience thought that they would enjoy using VR compared to those with no prior experience (*p* < 0.001).

### Willingness to use VR

Most participants reported willingness to use VR to manage their disease, regardless of their prior VR experience (85.1% for VR-naïve vs. 91.3% for VR-experienced participants) (Fig. 1). The most common ways in which participants were willing to use VR were for exercise, physical therapy, and education, all of which had a greater than 50.0% endorsement in both the VR-naïve and VR-experienced groups (Fig. 2).

## Discussion

Our team conducted a web-based survey of nearly 4000 people with rheumatologic conditions, a large proportion of whom had a diagnosis of RA. Most participants endorsed at least one area in which they would be willing to use VR to manage their disease. While only slightly more than a quarter of participants indicated that they thought VR would be useful to learn about their condition, less than 10% indicated that they did not think it would be useful, with more than half of participants being unsure. Attitudes were even more positive among those who reported prior experience using VR. Interestingly, willingness to use VR to manage their condition was high among those with no or very limited prior experience using it, even with a moderate degree of uncertainty around perceptions of usefulness and anticipated enjoyment of VR. This suggests that people with rheumatologic conditions are amenable to learning more about how VR works and its potential uses, indicating an educational opportunity around the use of immersive technologies in health care and the potential benefit of concerted efforts to socialize patients to the use of VR as an educational tool, including opportunities to try out the technology.

We found some differences in comparing study variables across recruitment samples, possibly due to underlying demographic differences in these two patient populations (Supplementary Tables S1 and S2 and Supplementary Fig. S1). As shown in Supplementary Table S1, members of the AP registry are younger, and a greater proportion are female and white compared with patients in the ENRGY database, potentially contributing to differences found in perceived usefulness, anticipated enjoyment, and willingness to use VR to manage their condition among the two survey groups. AP is a voluntary online patient community who tend to be more active in software applications and is therefore likely to be more tech-savvy and highly educated than the ENRGY sample of patients drawn from practice sites who may be less open to trying new technologies. Interestingly, the difference in willingness to use VR across recruitment source was greater in the VR-naïve group compared with the VR-experienced group. Prior use of VR may close the gap created by openness to use new technologies.

A primary goal of patient education is to raise awareness of the consequences of disease progression without treatment, and this is challenging to achieve through traditional risk communication approaches.^19^ Yet education has been shown to be a valuable resource to help patients manage their rheumatic disease. For example, in a study by Pablos et al., patients with RA valued information about limitations in physical functioning resulting from suboptimal treatment.^20^ Further, a patient decision aid incorporating information about RA progression in the absence of optimal treatment helped patients make treatment decisions. Moreover, in a study by Barton et al., patients with RA expressed a desire for clinicians to consider their quality of life goals, in which physical function is a key part, in making treatment recommendations.^21^

Patient education tools used to demonstrate the consequences of natural disease progression on physical functioning and elicit patients’ quality of life goals would assist health care providers and patients to set treatment goals collaboratively. A previous study demonstrated that VR is an engaging learning tool for patients with abdominal aortic aneurysms to understand their health status.^22^ Our study found that many patients with rheumatologic diseases perceived VR as a useful and potentially enjoyable educational tool and were willing to use VR to manage their disease, suggesting that VR may be an effective educational tool for this population. However, there may be unintended consequences of the use of VR for this purpose that should be considered, including physical effects such as cybersickness, and emotional effects, including distress from experiencing the consequences of disease progression.

VR has been used successfully in patients with fibromyalgia and has been well-accepted. In a small study of six women with fibromyalgia (mean age of 55 years), VR paired with cognitive behavioral therapy was shown to significantly reduce pain and depression and increase positive affect and use of healthy coping strategies.^9^ The authors also reported high levels of acceptance of and satisfaction with the VR component of the intervention. A similar study that focused on the activity management component of a cognitive behavioral therapy (CBT) plus VR intervention in 61 women with fibromyalgia showed significant improvements in disability, perceived quality of life, coping strategies, and a high degree of satisfaction with the VR aspect.^10^ A third study in 20 patients with RA, lupus, or fibromyalgia (mean age of 53 years) experienced significantly reduced pain and anxiety with the use of VR for guided meditation and biofeedback.^11^ These relatively small studies show support for the use of VR, mostly among patients with fibromyalgia. There is limited research on the acceptance of VR among patients with other rheumatologic conditions. A recently completed NIH-funded trial (NCT04933474) being conducted by some of our study team (J.C.) enrolled more than 300 patients with chronic pain and randomized them to a VR-based vs. traditional intervention to teach CBT skills. Approximately half of the participants had one or more rheumatic or musculoskeletal diseases (RMD), suggesting that patients with RMD are interested in VR-based interventions. The current study extends these findings of acceptability and willingness to use VR to a large group of individuals with various rheumatologic conditions in two slightly older samples.

Limitations of the study are inability to infer causality between prior experience with VR and attitudinal variables due to the cross-sectional nature of the study, and potential bias from self-report, including recall and social desirability bias. Additionally, though existing surveys were used to guide the development of our survey, the items were self-constructed using the TAM as a framework and were not pretested, which may have impacted validity of the results. Related to this limitation, our question asking the participant to indicate ways they may be willing to use VR may not have been an exhaustive list. Accordingly, those who indicated “none of the above” may actually be willing to use VR in other ways. Considering this, if we had asked the question “are you willing to use VR to manage your disease?” directly, frequencies of willingness may be even greater. Despite a large sample size, participants were recruited from an online patient-powered research network and a community rheumatology PBRN who had a valid email address and chose to participate in a VR-related survey. A large proportion of participants were female and white. Accordingly, the findings of this study may not be generalizable to populations with other rheumatologic diseases or who lacked email or chose not to respond. People who indicated that they did not think VR could help them or their condition in the primary survey, though a very small proportion (12%) of the total sample, were not invited to participate in this survey. By excluding those individuals, it is possible that degree of perceived usefulness and willingness to use VR as a tool to learn about and help manage their disease may have been inflated. We have noted in the results how the frequencies in perceived usefulness change when considering these people as not perceiving VR to be useful to learn about their condition. Future studies should purposively sample other population subgroups and those who are skeptical of the utility of VR as an educational tool to determine their level acceptance.

## Conclusion

Even among those with no prior experience with VR, people with rheumatologic conditions (mean age of 63 years) are willing to use VR to manage their disease state. The findings of this study could inform the development of a VR educational tool for people with rheumatologic disease to support treatment decision-making.

## Data Availability

The datasets generated and analyzed during the current study are available from the corresponding author upon reasonable request.

## Abbreviations Used

AP: Arthritis Power
ENRGY: Excellence Network in Rheumatology
PBRN: Practice-based Research Network
RMD: rheumatic and musculoskeletal diseases
TAM: Technology Acceptance Model
VR: virtual reality
